# Mating frequency estimation and its importance for colony abundance analyses in eusocial pollinators: a case study of *Bombus impatiens* (Hymenoptera: Apidae)

**DOI:** 10.1093/jee/toae178

**Published:** 2024-08-13

**Authors:** Sydney A Bird, Nathaniel S Pope, Carley M McGrady, Shelby J Fleischer, Margarita M López-Uribe

**Affiliations:** Intercollege Graduate Degree Program in Ecology, The Pennsylvania State University, University Park, PA 16802, USA; Temperate Tree Fruit and Vegetable Research Unit, United States Department of Agriculture, 5230 Konnowac Pass Rd, Wapato, WA 98951, USA; Department of Entomology, The Pennsylvania State University, University Park, PA 16802, USA; Department of Biology, Institute of Ecology and Evolution, University of Oregon, Eugene, OR 97403, USA; Department of Entomology, The Pennsylvania State University, University Park, PA 16802, USA; Department of Entomology, The Pennsylvania State University, University Park, PA 16802, USA; Intercollege Graduate Degree Program in Ecology, The Pennsylvania State University, University Park, PA 16802, USA; Department of Entomology, The Pennsylvania State University, University Park, PA 16802, USA

**Keywords:** monogamy, COLONY software, conservation, pollinator, mating frequency

## Abstract

The genus *Bombus* (bumble bees) includes approximately 265 species, many of which are in decline in North America and Europe. To estimate colony abundance of bumble bees in natural and agricultural habitats, sibship relationships are often reconstructed from genetic data with the assumption that colonies have 1 monandrous queen. However, some species such as the North American common eastern bumble bee (*Bombus impatiens* Cresson) can display low levels of polyandry, which may bias estimates of colony abundance based on monandrous sibship reconstructions. To accurately quantify rates of polyandry in wild and commercially mated queens of this species, we empirically estimated mating frequencies using a novel statistical model and genotypes from 730 bees. To genotype individuals, we used a highly polymorphic set of microsatellites on colonies established from 20 wild-caught gynes and 10 commercial colonies. We found multiple fathers in 3 of the wild colonies and 3 of the commercial colonies. This resulted in average effective mating frequencies of 1.075 ± 0.18 and 1.154 ± 0.25 for wild and commercial colonies, respectively. These findings agree with previous reports of low rates of polyandry for *B. impatiens*. Using a large empirical dataset, we demonstrate that assuming monandry for colony abundance estimation in species that violate this assumption results in an overestimation of the number of colonies. Our results emphasize the importance of studying mating frequencies in social species of conservation concern and economic importance for the accuracy of colony abundance estimation and for understanding their ecology and sociobiology.

## Introduction

The number of successful mates per female, known as mating frequency, helps inform the designation of a species’ mating system as monandrous (1 male mate) or polyandrous (multiple male mates). In social insect species, mating system is an important demographic parameter necessary for the estimation of colony abundance from genetic data (Jones and Wang 2010). For social insects, population size estimates derived from numbers of individuals counted in the wild can misrepresent key population-level processes because the colony is the reproductive unit. Thus, accurate estimation of colony numbers is crucial for species of conservation concerns, as it is used for the identification of drivers of species declines (e.g., correlating population status with land use history; Maebe et al. 2015), for the monitoring and eradication of invasive species (Toquenaga and Kokuvo 2010), and the development of plans for conservation actions and priorities (Hansen and Jensen 2005, Lepais et al. 2010, McGrady et al. 2021). Many social pollinator species are in decline, which underscores the importance of accurate estimations of colony abundance for these species (Powney et al. 2019).

For colony abundance estimations, sibship relationships between wild-collected workers are assigned based on multilocus genotypes. Under Mendelian inheritance, full-sibling groups are genetically distinct. For monandrous haplodiploid species, 1 full-sibling group represents 1 colony, and workers share on average 75% of alleles among themselves and 50% of the alleles with the queen (Hamilton 1964). Therefore, family relationships can be easily assigned based on genetic data (Wang 2004, Lepais et al. 2010, Toquenaga and Kokuvo 2010). However, in a polyandrous colony, there may be multiple full-sibling groups. In this case, the average relatedness among members of a colony decreases and more genetic markers are needed to improve the chances of distinguishing between half-siblings and full-siblings (Wang 2004, Wang and Santure 2009). For example, if 1 father produces 2 offspring, they will share on average 50% of the maternal alleles and 100% of the paternal alleles. However, if 2 unrelated fathers each produce 1 offspring, the half-siblings will only share maternal alleles and will not share any paternal alleles. Ultimately, by understanding the levels of polyandry typical of a species, we can better estimate true colony numbers within a population by inferring the numbers of full-sibling groups present in a population sample.

In addition to variation in mating frequencies, another complication that reduces the ability to distinguish between full- and half-siblings is the presence of genotyping error. Microsatellite markers—short simple repeat regions present throughout the genome—are among the most common markers used for sibship reconstruction because of their highly polymorphic nature (Ashley et al 2008, Li et al. 2013, Geib et al. 2015, Hama-Ali et al. 2015, McGrady et al 2021, Conflitti et al. 2022). These markers are prone to 2 types of errors: allelic dropout, in which 1 allele fails to amplify giving the appearance of a homozygous genotype, and mistyping errors, in which alleles are incorrectly identified or mistyped during genotyping (Wang 2004). These types of errors can mimic different alleles from different paternal lines and thus erroneously generate inference of full- or half-sibling groups. In the COLONY software (Wang 2004, Jones and Wang 2010), which many researchers use to calculate mating frequency, error rates per locus must be assigned a priori by the user. However, Bayesian approaches such as the methods proposed by Hadfield et al. (2006) allow for jointly estimating paternity and error rates.

**Fig. 1.**
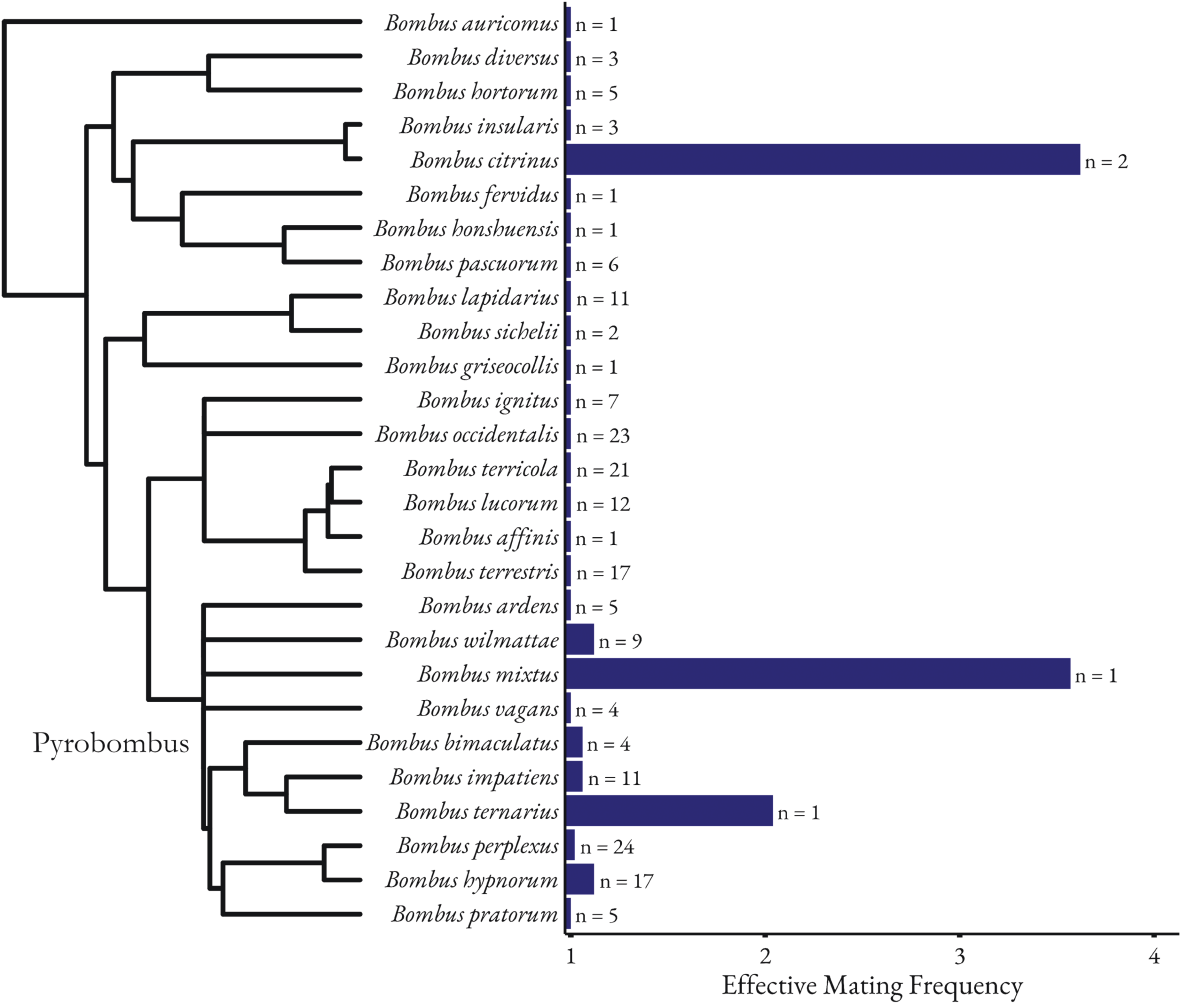
Reported estimates of mating frequency in the literature for species in the genus *Bombus*. A phylogenetic tree illustrating the relationships between species with available data on mating frequency is shown to the left of the y-axis. Phylogeny was adapted from Cameron et al. (2007). The x-axis shows the effective mating frequency as calculated by Starr (1984). Mating frequency of 1 represents a monandrous species. *Bombus* species are organized phylogenetically on the y-axis. Sample sizes (number of colonies evaluated) are shown next to bars. Mating frequencies are drawn from 7 studies with additional information and citations in Supplementary Material S1.

Bumble bees (genus *Bombus*) are a group of social and socially parasitic species of conservation concern that provide critical pollination services to a variety of crops (e.g., Goulson et al. 2008, Artz and Nault 2011, McGrady et al. 2021). While most bumble bee species are monandrous, some appear to deviate from this mating system (Fig. 1). Still, mating frequencies have been empirically studied for only about 10% of the approximately 265 species of bumble bees (Williams 2024), and in many cases, these estimates are derived from a small number of colonies and genetic markers (Supplementary Material S1). Within the subgenus *Pyrobombus*, low levels of polyandry have been reported in at least 7 species (Fig. 1). This subgenus (*n* = 43 species) includes key agricultural pollinators such as *Bombus impatiens* Cresson, a model for North American bumble bee research, as well as several other abundant and widespread bumble bee species (Cameron et al. 2007). Despite this, most studies estimating colony density in these species assume monandrous mating (Schmid-Hempel and Schmid-Hempel 2000, Takahashi et al. 2008, Conflitti et al. 2022).

Here, we focus on a new methodological approach to estimate mating frequency in *B. impatiens*, which is one of the most common and important wild pollinators in eastern North America (Strange and Tripodi 2019, McGrady et al. 2021). Because of its effectiveness as a pollinator and amenability to management, *B. impatiens* is commercially produced and used to provide pollination services in agricultural fields and greenhouses (Cnaani et al. 2002, Velthuis and Van Doorn 2006, Suni et al. 2017). Population monitoring of this species suggests that it is stable or increasing throughout its range compared to other North American bumble bee species (Cameron et al. 2011). One study evaluated mating frequencies in 11 colonies of wild *B. impatiens* and found 3 of those 11 colony’s queens mated with 3 males each while the rest of the colonies were monandrous, showing a higher level of polyandry compared to other *Bombus* species (Payne et al. 2003; Fig. 1). However, this information was based on few loci, a small number of colonies, and average effective mating frequency could not be calculated due to the unknown contribution of the proportion of offspring by each father.

In this study, we used microsatellite genotypes of queens and workers from colonies founded by wild-caught gynes (henceforth referred to as “wild” colonies) and commercial *B. impatiens* colonies to estimate the mating frequency of *B. impatiens* queens. We obtained genotypes from queen and worker bees from 20 wild and 10 commercial colonies using an optimized set of 11 microsatellite loci (Supplementary Material S2). In addition, we adapted the model implemented by the software COLONY (Wang 2004, Jones and Wang 2010) to create a Bayesian inference algorithm that jointly estimates paternity and genotyping error for each colony to increase the accuracy of kinship estimations. We then evaluated how assumptions of monandry or polyandry affect estimates of colony numbers based on inferred sibling groups using a previously published dataset of 6,306 worker genotypes from 30 agricultural sites across Pennsylvania (USA) (McGrady et al. 2021).

Our study provides 3 major findings that have important implications for estimating colony abundance in social bee pollinators: (i) accurate estimates of mating frequencies in wild and commercial *B. impatiens*; (ii) a statistical approach that can accurately determine mating frequencies for other social species while estimating genotyping error; and (iii) a demonstration that monandrous or polyandrous mating frequency assumptions can have a large impact on the results of colony abundance estimation from inferred sibships. Our statistical approach represents a valuable tool for studying other social species and determining their mating frequencies. By improving the accuracy of colony abundance estimates, our research contributes to better-informed conservation strategies and actions for social bee pollinators that will ultimately support their long-term survival and the vital pollination services to natural and agricultural ecosystems.

## Materials and Methods

### Specimen Collection and Rearing

Wild *B. impatiens* gynes were collected between April and May of 2018 from 3 locations in Pennsylvania, USA: University Park, Newport, and Landisville. Twenty colonies established from these wild-caught gynes were then reared following standard protocols (Treanore et al. 2021). Colonies were held in a walk-in incubator under constant darkness, at 28–30 °C, 60% RH, and supplied ad libitum with a 60% sucrose solution and honey bee collected pollen. Colony maintenance and offspring collection were performed under red light. To quantify mating frequencies in wild colonies, we collected the first 10 emerging workers, and if possible, 10 additional workers approximately 3–4 weeks after to capture any differences in paternity in the later emerging workers. The queen was removed and frozen along with the rest of the workers after collections of the second cohort. Additional bees with unknown emergence dates were also collected for a total of 13–32 bees per wild colony. For the assessment of mating frequencies in managed colonies, 10 commercial colonies were purchased from Koppert Biological Systems (Howell Michigan, USA). Commercial colonies were purchased and left in the field until late summer when the entire colony was removed from the field and frozen. The original queen and 14–21 workers per colony were removed for microsatellite genotyping analysis.

### Quality Assessment of Microsatellite Loci Population Genetic Analysis

We used 11 microsatellite markers previously developed for *B. impatiens* and other *Bombus* spp (Supplementary Material S2). Because the family relationships within our dataset violate assumptions of Hardy–Weinberg equilibrium, we used the genetic data published by McGrady et al. (2021) to estimate parameters of allele frequencies of the source population for the 11 loci included in this study. We calculated the number of alleles per locus and evaluated the probability of linkage disequilibrium using the package *poppr* (v2.9.3, Kamvar et al. 2014) in R Statistical Software (v4.1.2; R Core Team 2020). We performed null allele analysis according to Chakraborty et al. (1994) using the package *PopGenReport* (v3.0.7, Adamack and Gruber 2014). To evaluate deviations from Hardy–Weinberg equilibrium, we performed a test with chi-squared comparisons using the R package *pegas* (v1.1, Paradis 2010). Given that we included 11 loci for all tests, we used the corresponding Bonferroni adjusted α value of 0.0045 for Hardy–Weinberg analysis and 0.0009 for linkage disequilibrium to correct for multiple comparisons.

### Microsatellite Genotyping

After pinning, the right mesothoracic leg from each bee was removed and placed in a 96-well PCR plate for DNA extraction. A total of 150 μl Chelex 100 (5%, in milli-q H20) and 5 μl of 10 mg/ml Proteinase K were added to each well of the plate containing a leg. Each sample was then heated in a Mastercycler pro thermocycler for 60 min at 55 °C, 15 min at 99 °C, 1 min at 37 °C, and 15 min at 99 °C. Extracted DNA was stored for up to 3 weeks in a 4 °C refrigerator. We amplified 11 microsatellite loci with different concentrations optimized for multiplex amplification (Supplementary Material S2). Polymerase chain reactions were performed using a Mastercycler pro thermocycler with the following cycle settings: 3 min 30 s initial denaturation at 95 °C, followed by 30 cycles of 30 s at 95 °C, 1 min 15 s at an annealing temperature of 55 °C and 45 s at 72 °C, then a final 15-min extension at 72 °C with a hold temperature at 15 °C. Following amplification, PCR products were diluted 1:10 with molecular water, then sized on an ABI3730XL DNA Analyzer (Applied Biosystems) with GeneScan LIZ 500 internal size standard (Applied Biosystems) at the Penn State Genomics Core Facility.

### Paternity Reconstruction and Power Analysis

Fragment results were imported into Geneious Prime v.2022.0.1 (Biomatters Ltd) for genotyping and alleles were scored manually. Dropout and mistyping error rates were estimated jointly with the posterior probability of the number of fathers on a colony-by-colony basis. To jointly estimate paternity and error rates while including known queen genotypes, we developed a Bayesian approach to paternity reconstruction based on the genotyping error model in Wang (2004). Briefly, the model assumes that observed genotypes are perturbed from true genotypes by 2 classes of errors: dropouts (ɛ1 where a heterozygous genotype appears homozygous) and mistyping (ɛ2 where a given allele appears to be another allele). The likelihood is calculated by summing (integrating) over possible maternal, paternal, and offspring genotypes conditional on a paternity assignment. We used a nonparametric prior (a Dirichlet process) on full-sibling groups and developed a Gibbs sampler that targets the joint posterior distribution of error rates and paternity assignments. The model and Gibbs sampler are detailed in Supplementary Methods and are implemented in an R package (https://github.com/nspope/paternityDP). To assess the accuracy of the method under realistic conditions, we applied it to 100 simulations for each combination of number of fathers (1–6) and error rates (0.01, 0.05, and 0.1 for both classes of errors but consistent across loci). Each simulation used parental genotypes that were sampled from the observed maternal allele frequencies from wild colonies; and produced observed genotypes for 20 offspring and a mother. In each simulation, 1 father was assigned the majority of offspring and each additional father was allocated only 1 offspring, as this represents the most challenging conditions for distinguishing genotyping errors from distinct paternal genotypes. To determine the impacts of different numbers of loci on the accuracy in jointly estimating paternity and error rates, 100 simulations were repeated with 2, 4, 8, and 16 loci. To simplify the analysis, each locus was assigned a conservative number of 3 alleles at equal frequencies and a fixed error rate of 0.05 for both dropout and mistyping.

To reconstruct the paternity of our focal wild and commercial colonies, we applied our paternity reconstruction method to the empirical genotypes from each colony, after removing bees/loci with > 50% missing genotypes, monomorphic loci, and “drifter” bees. These “drifters” are individuals who originated from a different mother colony and likely escaped their original colony and returned to a different colony during colony maintenance procedures. Because the genotypes of the queens were known, the identification of drifters was accomplished by including a second unobserved queen to the model. After paternity reconstruction, we calculated effective mating frequency—defined as $\frac{1}{\sum p_{i}^{2}}$, where *p*_*i*_ is the proportion of each offspring that belongs to father *i* (Starr 1984)—by averaging over 1,000 Markov chain Monte Carlo samples for each colony. All following statistical tests were computed in R v.4.1.1 (R Core Team 2020).

### Colony Abundance Estimation From Sampled Worker Genotypes

To determine the influence of polyandrous or monandrous assumptions when recreating sibships from genetic data, we reanalyzed a previously published dataset of 6,306 *B. impatiens* workers collected from 30 agricultural field sites in Pennsylvania (USA) (McGrady et al. 2021) that used the same loci and approach for genotyping used in our study. For each site, the genotypes of the workers were imported into COLONY v.2.0.6.5 (Wang 2004, Jones and Wang 2010) to reconstruct sibship relationships among individuals. We analyzed the data twice in COLONY: once with the assumption of female monandry, and once with the assumption of female polyandry, as the software only provides a binary parameter for mating frequency. For monandry, the number of full-sibling groups was recorded as the number of detected colonies and the number of individuals comprising each colony was recorded. In order to simplify the analysis, we did not reject any full-sibling groups based on COLONY’s statistical probability of accuracy. For polyandry, the estimated number of mothers was recorded as the number of detected colonies, including half-siblings. In addition, the number of workers comprising each colony was also recorded. Mating frequencies were calculated from the output of COLONY with polyandry to compare it with our empirical estimation of mating frequencies in *B. impatiens*. A Wilcoxon rank-sum test was used to determine whether mating frequency differed between monandrous and polyandrous colonies. Total colony abundance was estimated in R with the package CAPWIRE (Miller et al. 2005) for both monandrous and polyandrous colonies from each site. A Welch’s paired *t*-test was performed in R v.4.1.1 (R Core Team 2020) to determine if the number of detected and estimated colonies differed when calculated under assumptions of monandry and polyandry.

## Results

### Summary of Microsatellite Diversity and Amplification Metrics

Due to the nature of our data set which contained family units, parameters of genetic diversity from our set of microsatellites were calculated using the data set of 6,306 field-collected individual worker genotypes of *B. impatiens* (McGrady et al. 2021). Within this data set, of the 11 analyzed loci, all were polymorphic with more than 5 alleles per locus. Only 1 locus (BTMS0081) deviated significantly from Hardy–Weinberg equilibrium (χ² = 636.961, *df* = 36, *P* = 0.001) (Table 1). There was no evidence of null alleles at any of the loci (Table 1). Linkage disequilibrium analysis revealed no significant association between different loci (*Ia* = 0.221, *P* > 0.0009).

**Table 1.** Results of estimations of locus genetic diversity of the McGrady et al. (2021) data set. Locus name, χ² value, degrees of freedom, *P*-value, confidence intervals, and observed and median frequencies for null allele analysis according to Chakraborty et al. (1994). Asterisks (*) indicate loci with significant deviations from Hardy–Weinberg equilibrium or presence of null alleles.

	Hardy–Weinberg			Null allele		
Locus	χ²	*df*	*P*-value	95% CI	Observed	Median
BTMS0066	466.740	465	0.022	0.00–0.02	0.01	0.01
B124	611.418	630	0.166	0.00–0.01	0.00	0.00
Btern01	307.446	351	0.140	−0.01–0.00	0.00	0.00
BT28	1.327	6	0.450	0.00–0.02	0.00	0.00
BTMS0062	1,673.300	1540	0.037	0.00–0.01	0.00	0.00
BTMS0073	13.784	15	0.134	0.00–0.02	0.01	0.01
BT10	398.482	406	0.029	0.00–0.01	0.00	0.00
BL11	282.292	231	0.029	0.00–0.01	0.00	0.00
BT30	57.948	91	0.243	−0.01–0.01	0.00	0.00
B96	416.275	378	0.293	−0.01–0.00	0.00	0.00
BTMS0081	636.961	36	0.001*	−0.01–0.01	0.00	0.00

For the mating frequency calculation, we genotyped a total of 501 bees from 20 wild colonies and 229 bees from 10 commercial colonies. Workers were discarded from analysis if genotypes were missing for more than 50% of loci. Loci with greater than 50% missing genotypes were removed from analyses on a colony-by-colony basis. Of the 11 loci analyzed, locus BT28 was entirely monomorphic within these individuals and removed from all subsequent analyses. Of the remaining loci, 9 were highly polymorphic with 4 alleles or more per locus (Supplementary Material S2). Each colony was evaluated with no fewer than 6 polymorphic loci, following the minimum set by Lepais et al. (2010). Due to data quality and insufficient number of workers, colony 13 was only evaluated with 3 polymorphic loci and was removed from subsequent analyses. Estimated genotyping error rates across colonies were low. Specifically, dropout error (ɛ1) ranged from 0.0025 to 0.00282 (ɛ1 median 0.0094) and mistyping error (ɛ2) ranged from 0.0027 to 0.0476, among colonies (ɛ2 median 0.0092) (Supplementary Material S4).

### Method Validation Through Power Analysis

**Fig. 2.**
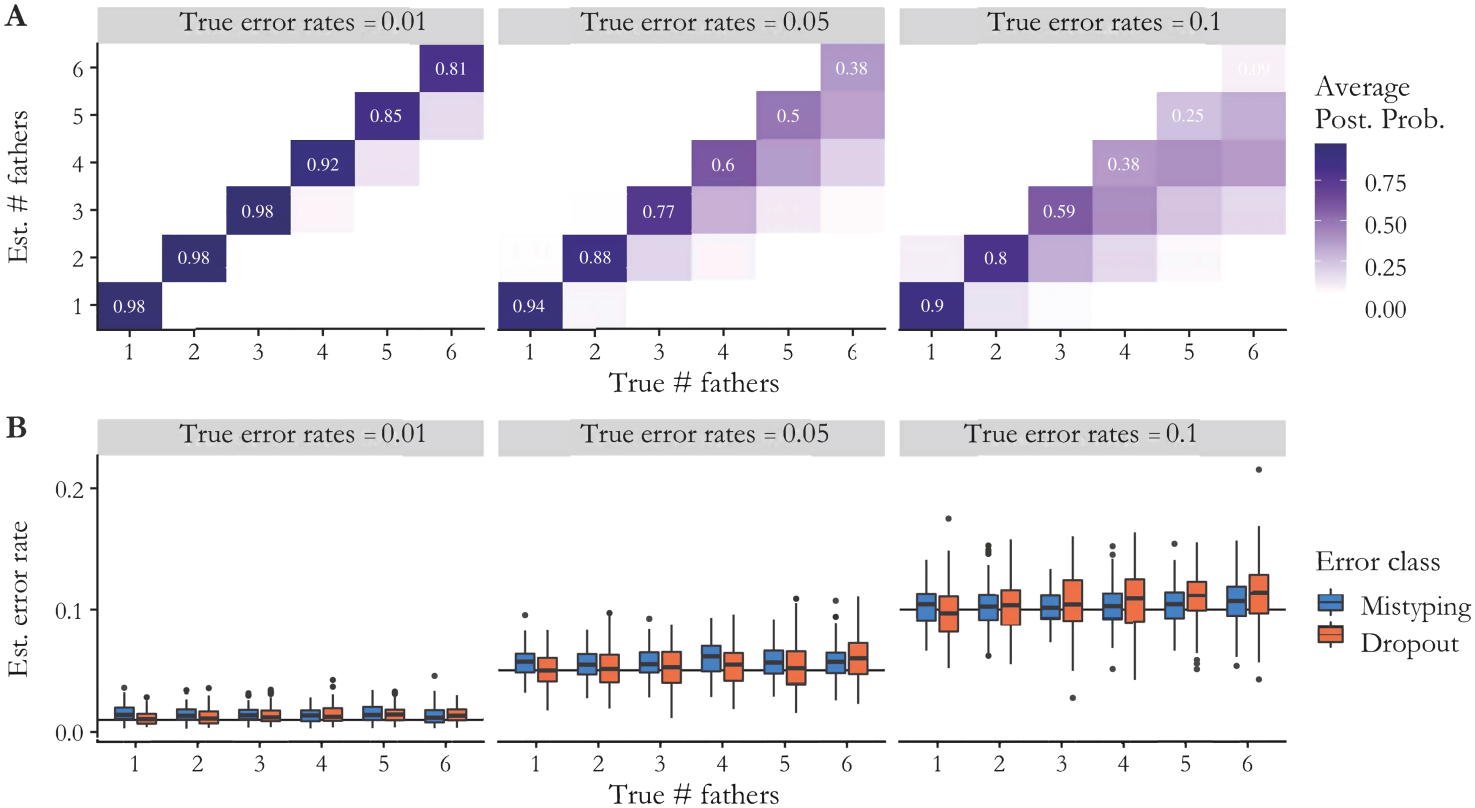
Accuracy of paternity reconstruction method for estimating number of fathers and genotyping error rates on simulated data. A) Average posterior probabilities (over 100 simulated colonies) for the estimated number of fathers (y-axis) given the true number of fathers (x-axis). Note each “column” sums to 1. Additional fathers beyond the first are only assigned 1 offspring (out of 20 total offspring/simulation) to emulate high paternity skew. The method is accurate for low error rates, but at high error rates cannot distinguish many fathers (> 3–4) from errors. B) Estimated genotyping error rates across simulations. For each class of errors, a global rate was used across loci. Boxplots show the distribution of the posterior mean across simulations. Regardless of the true number of fathers, error rates are estimated accurately.

**Fig. 3.**
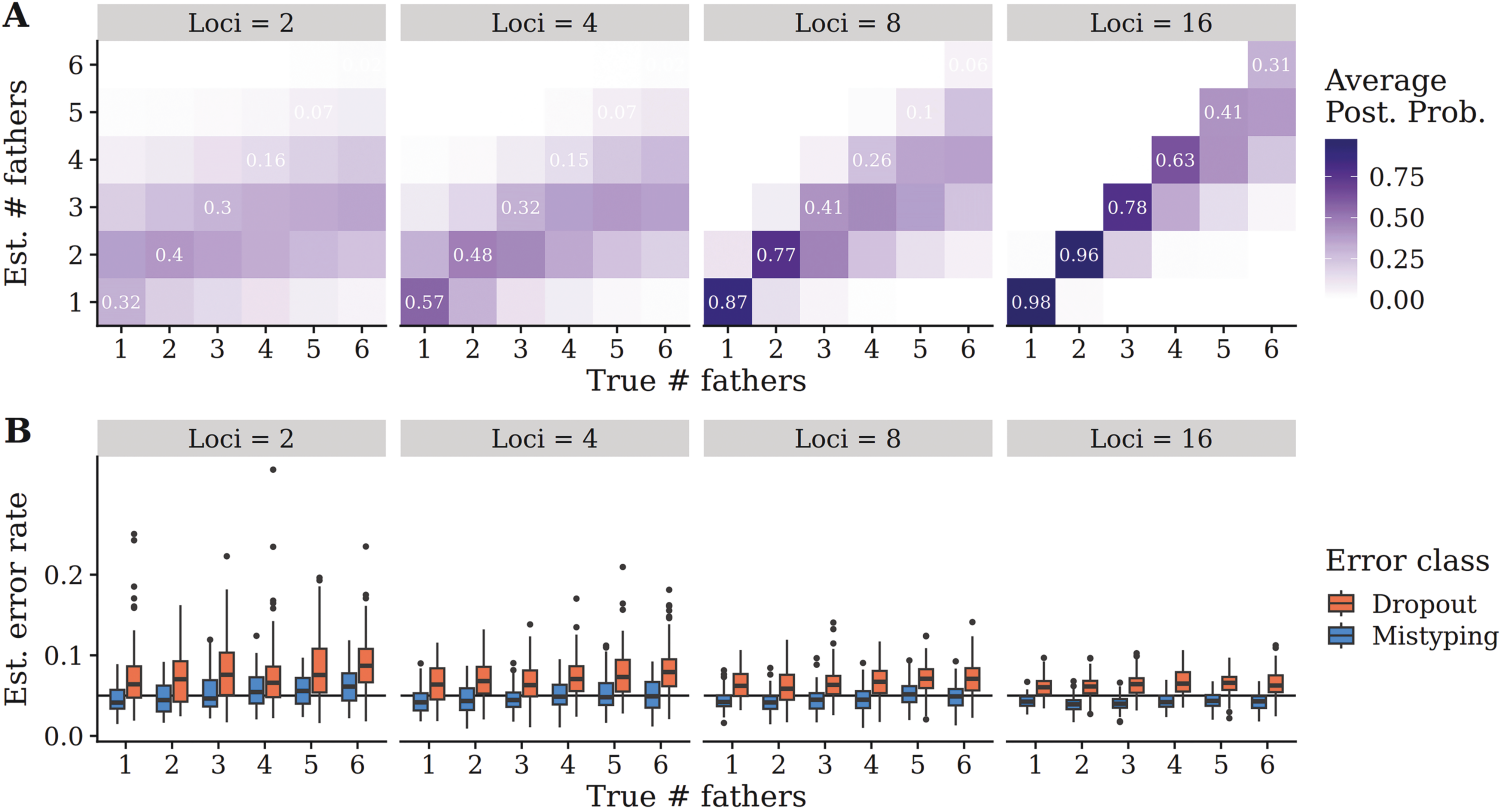
Accuracy of paternity reconstruction method for estimating number of fathers and genotyping error rates on simulated data with different numbers of loci. A) Average posterior probabilities (over 100 simulated colonies) for the estimated number of fathers (y-axis) given the true number of fathers (x-axis) with 2, 4, 8, and 16 loci. Additional fathers beyond the first are only assigned one offspring (out of 20 total offspring/simulation) to emulate high paternity skew. B) Estimated genotyping error rates across simulations with different number of loci. For each class of errors, a global rate of 0.05 was used across loci. Boxplots show the distribution of the posterior mean across simulations.

To demonstrate the accuracy of our paternity reconstruction method, we performed a power analysis using colonies simulated using the observed maternal allele frequencies at the 11 microsatellite loci (Figs. 2 and 3). These simulations illustrated that the accuracy for the estimated number of fathers was above 80% for 1–2 fathers regardless of either mistyping or dropout error rate (Fig. 2A) and the accuracy for detecting arbitrary numbers of fathers was also above 80% when error rates were low (ɛ1 = 0.01 and ɛ2 = 0.01). When error rates were high (ɛ1 and ɛ2 = 0.1), the model did not distinguish high numbers of fathers (> 3) from genotyping errors. Furthermore, error rate estimates were accurate regardless of the number of fathers (Fig. 2B). Power to distinguish monandry versus polyandry was high regardless of both the true number of fathers and the error rate. Power to predict polyandry and accurately estimate error rate increased with increasing numbers of loci (Fig. 3). With moderate error rates of 0.05, at least 8 loci were needed to distinguish monandry from polyandry with certainty. With few loci, the estimated dropout rate was inflated; however, the estimated mistyping rate remained fairly accurate. With 16 simulated loci, the algorithm had very high accuracy in distinguishing between 1 and 2 fathers even at moderate error rates (Fig. 3).

### Paternity Reconstruction

**Fig. 4.**
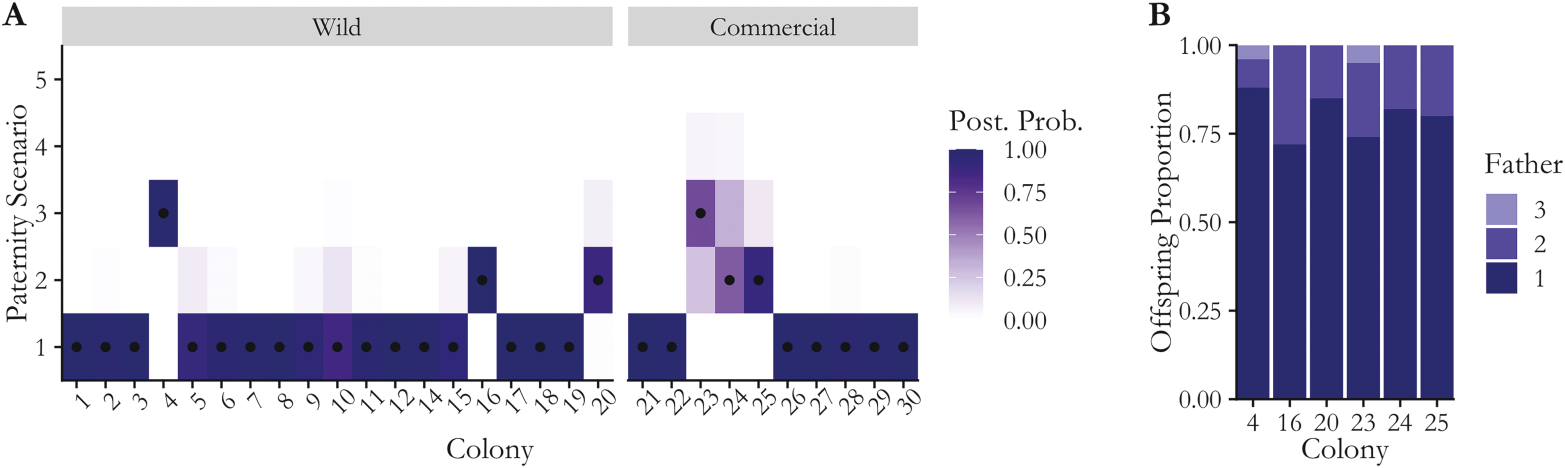
Results of paternity estimation for 20 wild and 10 commercial *B. impatiens* colonies. A) Paternity estimates for each colony showing the posterior probabilities for the possible number of different fathers on a gradient based on the accuracy of each paternity scenario per colony. The best estimate of the true number of fathers represented in each colony is marked with a black dot. Average effective mating frequency for wild colonies was 1.07 and for commercial colonies was 1.15. B) Progeny contribution of each father in polyandrous colonies showing that one father contributes a majority proportion of offspring in polyandrous colonies. The x-axis shows each polyandrous colony. The proportion of offspring is shown on the y-axis and bars are shaded based on which father contributed each proportion of offspring.

Of the 19 colonies founded by wild-caught gynes, we found 16 monandrous colonies. Of those that were polyandrous, we found 2 colonies with 2 fathers and 1 colony with 3 fathers (Fig. 4A). Of the 10 commercial colonies, 7 were monandrous, 2 showed 2 fathers, and 1 showed 3 fathers within the sibling set. Contributions of the second or third father to the offspring pool were less than 30% of the progeny (Fig. 4B). Average effective mating frequency was 1.075 ± 0.18 for wild colonies and 1.154 ± 0.25 in commercial colonies. A Wilcoxon rank-sum test showed that wild and commercial colony effective matings were not significantly different (*W* = 102, *P* = 0.38). In 9/20 wild colonies and 5/10 commercial colonies, we identified 1–6 individuals that were not related to the original queen, suggesting the presence of drifters. We removed a total of 17 drifter bees from these commercial colonies and 15 drifters from the wild colonies (Supplementary Material S5). In all wild colonies except colony 3 and colonies 17–20, workers were sampled temporally and separated into early emerging and later emerging. In colony 16 and colony 4, the offspring of the minority fathers were all in the second (later emerging) cohort of workers. However, this was not the case in the other polyandrous colonies.

**Fig. 5.**
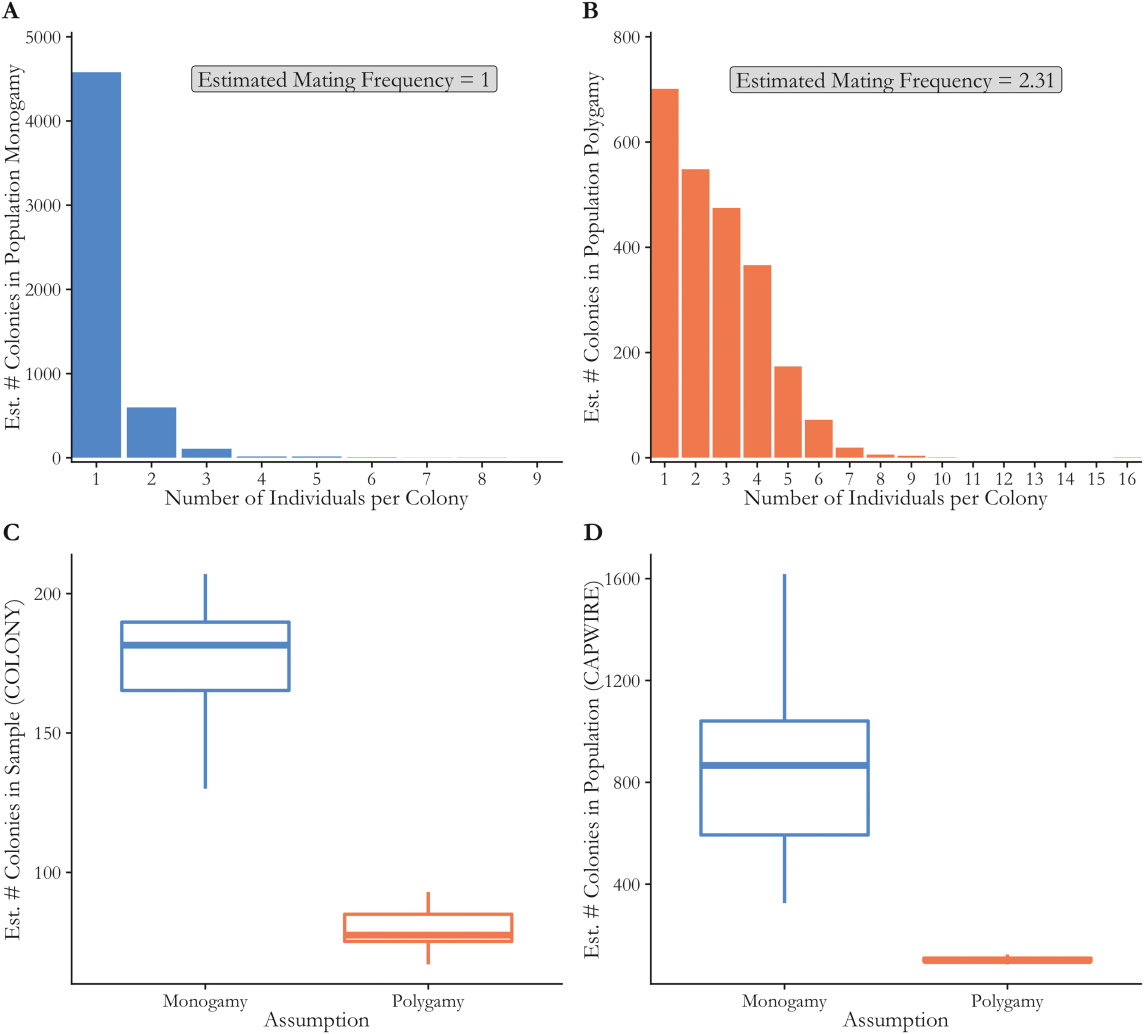
Testing the effect of the monandry assumption on estimates from COLONY and Capwire from a reanalysis of field-collected workers from 30 agricultural field sites. A and B) Histograms show the frequency of the number of individuals per colony detected in COLONY under the assumption of monandry A) and polyandry B). C) Boxplots showing the number of detected colonies by COLONY using assumptions of monandry or polyandry D) Boxplot showing the colony abundance per site estimated by CAPWIRE using the 2 outputs from COLONY. Note that the polyandry assumption in COLONY resulted in an effective mating frequency of 2.31, higher than our observed value of 1.07, contributing to the large discrepancy in estimates of colony abundance.

From the previously published genotypes of 6,306 bees collected from 30 agricultural field sites, we found that the average number of detected full-sibships (representing colonies) per site was greater using the assumption of monandry (177 full-sibling groups, representing colonies) than using the assumption of polyandry (79 inferred queens, representing colonies) (*df* = 35.87, *P* < 0.001) (Fig. 5C). The estimated effective mating frequency based on the COLONY output for polyandry was 2.31. Similarly, the total estimated number of colonies derived from CAPWIRE was greater when monandry was the chosen COLONY parameter (861 colonies per site) than when polyandry was the chosen COLONY parameter (102 colonies per site) (*df* = 29.057, *P*-value < 0.001) (Fig. 5D). The inflated numbers of total estimated monandrous colonies compared to total polyandrous colonies were driven by an excess of singleton colonies (colonies represented by only 1 detected worker) (Fig. 5A and B). When assuming monandry, singleton colonies were by far the most common, compared to polyandry where singleton and doubleton colonies are more equally represented (Fig. 5A and B).

## Discussion

Here, we estimated the effective mating frequency of wild and commercial *B. impatiens* colonies using a novel statistical method that jointly estimates paternity and error rates using 10 polymorphic microsatellite markers. While the environments for wild versus commercial mating are very different, here we found that the effective mating frequencies were not significantly different (1.075 and 1.154, respectively). The estimated genotyping error rates were low across colonies (median error rate for all colonies > 0.01 for both dropout and mistyping). We verified the accuracy of our statistical method through a power analysis that demonstrated that this algorithm can accurately estimate both the number of effective mates per queen and dropout and genotyping error. We also demonstrated that colony abundance estimations from genetic data are very sensitive to mating system assumptions. The COLONY software is currently limited to a binary mating frequency parameter, so users must choose between female monandry and polyandry. When we tested the differences in colony abundance between these 2 parameters, we found that the number of detected and estimated colonies in the population was twice as high when using the assumption of monandry than polyandry. Due to the highly biased contributions of each father in polyandrous colonies, *B. impatiens* still has mating frequencies very close to monandry despite violations of this assumption in 3 out of 19 of the analyzed wild colonies and 3 out of 10 commercial colonies.

The accurate estimation of mating frequency in eusocial pollinators is important for their conservation and understanding their ecology and sociobiology. Despite this, for bumble bees, we found that only 27 of the 265 *Bombus* species (Williams 2024) have been evaluated for mating frequency. We found that 7 of those species show deviations from monandry, particularly within the subgenus *Pyrobombus* (Fig. 1). Due to findings of complete genetic monandry in most *Bombus* species, many studies have assumed monandry when reconstructing sibships from genetic data in *Bombus* (Schmid-Hemple 2000; Takahashi et al. 2008, Jha and Kremen 2013, McGrady et al. 2021, Timberlake et al. 2021, Conflitti et al. 2022). Our results demonstrate that assuming monandry for colony abundance estimation in *Bombus* may lead to a slight overestimation of the number of colonies. This overestimation of colony abundance could lead to flawed decisions for the protection of species that may be of conservation concern (Carvell 2002, Goulson et al. 2008, Memmott et al. 2010). Similarly, estimates of the number of bumble bee colonies providing pollination services in agroecosystems may be overestimated leading to erroneous conclusions about the abundance and resilience of wild bumble bees that inhabit agroecosystems (McGrady et al. 2021). However, we also show that the estimated mating frequency in the polyandrous COLONY reconstruction was significantly higher than the actual mating frequency of *B. impatiens* (2.31 in COLONY versus 1.07 actual). Therefore, choosing the polyandrous mating parameter may lead to an underestimation of colony abundance in lowly polyandrous species. Our data support that using monandrous assumptions is closer to the actual mating frequencies in *B. impatiens* than the polyandrous assumption. Future iterations of sibship reconstruction software (such as COLONY) should consider the incorporation of a mating frequency parameter in which the actual mating frequency of the species can be used in the estimations.

Accounting for genotyping errors is crucial for accurate sibship reconstruction for methodologies to distinguish between half-siblings and erroneous genotypes. Through a power analysis, we showed that our modified statistical method is robust for distinguishing 1–3 fathers with up to 0.05 genotyping error rate, and has greater than a 90% chance of accurately determining 1 father versus multiple fathers when error rates are less than 0.1 (Fig. 2A). The estimated genotyping errors of the loci in our dataset were lower than 0.01, which is within the range of genotyping errors that allow the sibship reconstruction algorithm to work accurately. When error rates are low (~0.01), the algorithm has a 90% chance of accurately determining the number of fathers for workers from polyandrous queens that mated between 2 and 4 times. The ability to accurately detect the number of fathers was limited to above 5 fathers, especially as error rates increased. In colonies 1, 5, 15, and 16, the queen could not be collected and genotyped. However, in these cases, we were able to reliably infer queen genotypes from her offspring and retain high posterior probabilities in the parentage estimate (Fig. 4). While we found that the power to differentiate monandry from polyandry was high, we did not model the possible scenario in which queens from each colony have the potential to be related to each other. If this analysis were conducted in species where there is potential for polyandry and sister queens that can mate with the same male, there may be decreased power for determining mating frequency. Our power analysis based on simulated data demonstrated that we can accurately estimate median genotyping error rates without empirical estimates regardless of the number of estimated fathers present in the colony (Fig. 2B). For colonies founded by wild gynes, 1 colony was evaluated on 10 loci, 1 was evaluated on 9 loci, 9 were evaluated on 8 loci, and 8 were evaluated on 7 loci (Supplementary Material S5). For commercial colonies, 2 were evaluated on 10 loci, 3 were evaluated on 9 loci, 4 were evaluated on 8 loci, and 1 was evaluated on 6 loci. However, the power analysis indicates that even with moderate error rates and low polymorphism, 8 loci are adequate for distinguishing monandry versus polyandry (Fig. 3A). Given that our estimated error rates were low, and polymorphism was high, we are confident that the paternity estimation was accurate for these colonies whose paternity estimation was based on fewer than 8 loci.

Our method also allowed us to accurately detect and remove individual bees identified as “drifters” that were unrelated to the original queen. These drifters likely escaped from their original colony and rejoined a different colony during the process of colony care and sampling workers in the walk-in growth incubator. Finding individuals that are unrelated to the queen is common in the wild and has been observed in various social species including bumble bees (Zanette et al. 2014, MacKenzie et al. 2021). If these drifters were not removed, this would erroneously increase mating frequency estimations within colonies. Given that we were able to identify and remove drifters from our dataset, the high polymorphism in many of our loci, and the low estimated error rates for our loci, we can confidently conclude that the accuracy of our sibship reconstruction and paternity estimates were high, and that *B. impatiens* shows low levels of polyandry.

In general, it is assumed that species in the genus *Bombus* are mostly monandrous (unlike honey bees, genus *Apis*) because multiple mating decreases the average relatedness within colonies, which in turn reduces colony cooperation due to weaker levels of kin selection (Boomsma 2009). Even though monandry increases the genetic relatedness among individuals in a colony and facilitates kin selection through increased inclusive fitness (Arnqvist and Nilsson 2000, Boomsma 2009, Fromhage and Kokko 2011), the low genetic diversity in monandrous colonies increases susceptibility to pathogens and decreases colony survival (Baer and Schmid-Hempel 1999, 2001, 2003). This cost of reductions in genetic diversity may outweigh the benefits to kin selection in certain eusocial species, resulting in the evolution of polyandry or multiple female mating (Boomsma 2009). Thus, some social species have secondarily evolved polyandry as observed in honey bees and some stingless bees (Neumann et al. 1999, Paxton et al. 1999, Tarpy et al., 2015). In addition, increasing colony genetic diversity may have other benefits to the colony through reductions of parasitism (Shykoff and Schmid-Hempel 1991, Baer and Schmid-Hempel 1999, 2001, 2003, Tarpy 2003), sex ratio conflict (Ratnieks and Boomsma 1995), diploid male production (Tarpy and Page 2002), and increases in the efficiency of worker performance (Fuchs and Moritz 1999, Jones et al. 2004) and tolerance to variable climatic conditions (Crozier and Page 1985). Despite the potential costs of monandry for bees, this mating system predominates in bumble bees. Our findings of low mating frequencies in commercial colonies—in settings where multiple mating is encouraged—suggest that there are some specific costs or barriers to multiple mating in bumble bees that remain to be characterized. One plausible scenario is that bumble bees mate multiple times in situations when the first mating event was not fully successful. However, all of these scenarios remain to be studied and characterized.

In this study, we confirm that *B. impatiens* exhibits a strong paternity skew meaning that in colonies established from polyandrous queens, 1 father contributed to the majority of the offspring compared to other mates. We found that 70% or more of the offspring in these colonies shared 1 father (Fig. 4B). This strategy is used by primitively eusocial species to maintain high intracolony relatedness while increasing genetic diversity through the development of progenies with high paternity skews (Jaffé et al. 2012). Mechanisms proposed to explain paternity skew in eusocial bees include cryptic female choice, which implies that queen bees can influence which sperm is used after copulation, and sperm competition (Eberhard 1996, Baer 2005). However, strategies for sperm storage and selection in bees, especially in non-*Apis* species, are relatively understudied. Franck et al. (1999, 2002) found that due to the deterioration in sperm clumping over time, *Apis mellifera* queens have more variation in the contribution to offspring of each father directly after mating, and as time goes on the contributions become more equal as sperm mixes. However, studies of paternity in highly polyandrous *A. mellifera* queens have found that queens use the sperm of all mates in representative proportions, with no bias toward the first or last mate (Laidlaw and Page 1984, Schlüns et al. 2005). Due to these findings, the alternative mechanism of “genotype scrambling” has been suggested for *Apis*, in which after discarding any unwanted ejaculate, queens may purposefully mix sperm, which increases genetic diversity of the progeny (Boomsma and Ratnieks 1996). Patterns of sperm mixing reported in *Apis* contrast our findings and other studies of *Bombus* that report a large bias of paternity toward a particular male. For instance, Huth-Schwarz et al. (2011) found that the dominant male in *Bombus willamette* sired between 71% and 82% of the offspring. Our data also showed that in 2 out of the 3 wild polyandrous colonies that were sampled temporally, offspring of the secondary father were only present in the second-generation worker cohort. It is possible that this was due to females running out of the first-mated male’s sperm, and using the sperm of the second- or third-mated male to fertilize the later-season offspring. However, due to the small sample size of polyandrous colonies, we are unable to determine whether the paternity skews occur by cohorts or seasonally. Future studies that evaluate mating frequency in *Bombus* should also sample workers that emerge at multiple timepoints to determine how paternity or paternity skew varies throughout the queen and colony’s lifecycle.

In summary, we confirm low levels of polyandry and strong paternity skews in *B. impatiens* using (i) a large sample size of wild and commercial colonies, (ii) a large set of microsatellite markers with low estimated error rates, and (iii) a new and more accurate statistical methodology for paternity reconstruction. The combination of our results and the implementation of a new methodology for determining mating frequency in social insects highlights the power of jointly estimating paternity and genotyping error when reconstructing sibship relationships. By demonstrating that the assumptions of either monandry or polyandry can have a significant impact on colony estimations, our results call attention to the need to incorporate mating frequency into statistical approaches that estimate colony abundance from genetic data rather than relying on a binary mating system parameter. Our results also highlight interesting areas for future research including the mechanisms that may sustain low levels of polyandry under both wild and commercial conditions and of sperm competition and drifting behaviors in *Bombus*.

## Supplementary Data

Supplementary data are available at *Journal of Economic Entomology* online.

toae178_suppl_Supplementary_Materials

## Data Availability

Data from this study are available from the Dryad Digital Repository:[doi:10.5061/dryad.pg4f4qrzk] (Bird 2024).
