# Supplementary material for: Mating frequency estimation and its importance for colony abundance analyses in eusocial pollinators: a case study of Bombus impatiens (Hymenoptera: Apidae)
Source: J Econ Entomol. 2024 Aug 13;117(5):1712–22. doi: 10.1093/jee/toae178 (PMC11646103; doi:10.1093/jee/toae178)
Supplement: toae178_suppl_Supplementary_Materials [file toae178_suppl_supplementary_materials.zip › Supp.Material1_Birdetal.docx]

Supplementary Materials 1

For “Mating frequency estimation and its importance ...” by Bird et al.

1. **Table of Effective Mating Frequencies in *Bombus***. Table shows species, reported mating frequency, the sample size or number of colonies evaluated, the reference study, and the number of microsatellite loci that were used to reconstruct sib-ships

| Species | Mating Frequency | SampleSize | Citation | Loci |
| --- | --- | --- | --- | --- |
| *Bombus mixtus* | 3.57 | n = 1 | Payne et al., 2003 | 1 microsatellite loci |
| *Bombus pratorum* | 1 | n = 5 | Schmid-Hempel & Schmid-Hempel 2000 | 4-5 microsatellite loci |
| *Bombus ardens* | 1 | n = 5 | Kokuvo et al., 2009 | 6 microsatellite loci |
| *Bombus hypnorum* | 1.12 | n = 17 | Schmid-Hempel & Schmid-Hempel 2000 | 4-5 microsatellite loci |
| *Bombus perplexus* | 1.02 | n = 24 | Owen et al., 2013 | 4 microsatellite loci |
| *Bombus ternarius* | 2.04 | n = 1 | Payne et al., 2003 | 1 microsatellite loci |
| *Bombus impatiens* | 1.06 | n = 11 | Cnaani et al., 2002* | 3 microsatellite loci |
| *Bombus wilmattae* | 1.12 | n = 9 | Huth-Schwarz et al., 2011 | 5 microsatellite loci (4 additional in one colony) |
| *Bombus bimaculatus* | 1.06 | n = 4 | Payne et al., 2003 | 1 microsatellite loci |
| *Bombus vagans* | 1 | n = 4 | Payne et al., 2003 | 1 microsatellite loci |
| *Bombus occidentalis* | 1 | n = 23 | Owen et al., 2013 | 4 microsatellite loci |
| *Bombus terricola* | 1 | n = 21 | Owen et al., 2013 | 4 microsatellite loci |
| *Bombus lucorum* | 1 | n = 12 | Schmid-Hempel & Schmid-Hempel 2000 | 4-5 microsatellite loci |
| *Bombus affinis* | 1 | n = 1 | Payne et al., 2003 | 1 microsatellite loci |
| *Bombus terrestris* | 1 | n = 17 | Schmid-Hempel & Schmid-Hempel 2000 | 4-5 microsatellite loci |
| *Bombus ignitus* | 1 | n = 7 | Takahashi et al., 2008 | 4 microsatellite loci |
| *Bombus griseocollis* | 1 | n = 1 | Payne et al., 2003 | 1 microsatellite loci |
| *Bombus sicheli* | 1 | n = 2 | Schmid-Hempel & Schmid-Hempel 2000 | 4-5 microsatellite loci |
| *Bombus lapidarius* | 1 | n = 11 | Schmid-Hempel & Schmid-Hempel 2000 | 4-5 microsatellite loci |
| *Bombus honshuensis* | 1 | n = 1 | Kokuvo et al., 2009 | 6 microsatellite loci |
| *Bombus pascuorum* | 1 | n = 6 | Schmid-Hempel & Schmid-Hempel 2000 | 4-5 microsatellite loci |
| *Bombus fervidus* | 1 | n = 1 | Payne et al., 2003 | 1 microsatellite loci |
| *Bombus citrinus* | 3.62 | n = 2 | Payne et al., 2003 | 1 microsatellite loci |
| *Bombus insularis* | 1 | n = 3 | Payne et al., 2003 | 1 microsatellite loci |
| *Bombus hortorum* | 1 | n = 5 | Schmid-Hempel & Schmid-Hempel 2000 | 4-5 microsatellite loci |
| *Bombus diversus* | 1 | n = 3 | Kokuvo et al., 2009 | 6 microsatellite loci |
| *Bombus auricomus* | 1 | n = 1 | Payne et al., 2003 | 1 microsatellite loci |

*Study was performed in commercial colonies

1. **References**

Cnaani J, Schmid-Hempel R, Schmidt JO (2002) Colony development, larval development and worker reproduction in *Bombus impatiens* Cresson. Insectes Sociaux *49*(2):164-170. https://doi.org/10.1007/s00040-002-8297-8

Kokuvo N, Toquenaga Y, Goka K (2009) Effective paternity in natural colonies of Japanese native bumble bees. Ecological research 24(5):1111-1115.

Owen RE, Whidden TL (2013) Monandry and polyandry in three species of North American bumble bees (*Bombus*) determined using microsatellite DNA markers. Canadian journal of zoology 91(7):523-528. https://doi.org/10.1139/cjz-2012-0288

Payne CM, Laverty TM, Lachance MA (2003) The frequency of multiple paternity in bumble bee (*Bombus*) colonies based on microsatellite DNA at the B10 locus. Insectes Sociaux, 50(4):375-378. https://doi.org/10.1007/s00040-003-0692-2

Schmid-Hempel R, Schmid-Hempel P (2000) Female mating frequencies in *Bombus* spp. from Central Europe. Insectes Sociaux 47(1):36-41. https://doi.org/10.1007/s000400050006

Takahashi JI, Itoh M, Shimizu I, Ono M (2008) Male parentage and queen mating frequency in the bumblebee *Bombus ignitus* (Hymenoptera: Bombinae). Ecological research, 23(6):937-942. https://doi.org/10.1007/s11284-007-0456-y
