# Supplementary material for: Mating frequency estimation and its importance for colony abundance analyses in eusocial pollinators: a case study of Bombus impatiens (Hymenoptera: Apidae)
Source: J Econ Entomol. 2024 Aug 13;117(5):1712–22. doi: 10.1093/jee/toae178 (PMC11646103; doi:10.1093/jee/toae178)
Supplement: toae178_suppl_Supplementary_Materials [file toae178_suppl_supplementary_materials.zip › Supp.Material2_Birdetal.docx]

Supplementary Materials 2

For “Mating frequency estimation and its importance ...” by Bird et al.

1. **Table of Microsatellite Loci Used for Sib-Ship Reconstruction.** Table shows the name of the loci, reference study, primer sequence, number of alleles, and observed heterozygosity for each loci.

*All primers had annealing temp of 55C

| Microsat | Ref | Primer Sequence | Alleles | H_o_ |
| --- | --- | --- | --- | --- |
| BTMS0066 | Stolle et al, 2009 | F: CATGATGACACCACCCAACG  R: TTAACGCCCAATGCCTTTCC | 10 | 0.736 |
| B124 | Estoup et al, 1995 | F: GCAACAGGTCGGGTTAGAG  R: CAGGATAGGGTAGGTAAGCAG | 17 | 0.857 |
| Btern01 | Funk et al, 2006 | F: CGTGTTTAGGGTACTGGTGGTC  R: GGAGCAAGAGGGCTAGACAAAAG | 13 | 0.707 |
| BT28 | Funk et al, 2006 | F: TTGCTGACGTTGCTGTGACTGAGG  R: TCCTCTGTGTGTTCTCTTACTTGGC | 1 | 0 |
| BTMS0062 | Stolle et al, 2009 | F: CTGTCGCATTATTCGCGGTT  R: CTGGGCGTGATTCGATGAAC | 24 | 0.808 |
| BTMS0073 | Stolle et al, 2009 | F: CGATATCGCGATCTTCGTACAC  R: GTAGCATGCTCTCCGTGTTG | 4 | 0.236 |
| BT10 | Funk et al, 2006 | F: TCTTGCTATCCACCACCCGC  R: GGACAGAAGCATAGACGCACCG | 15 | 0.804 |
| BL11 | Funk et al, 2006 | F: AAGGGTACGAAATGCGCGAG  R: TGACGAGTGCGGCCTTTTTC | 12 | 0.764 |
| BT30 | Funk et al, 2006 | F: ATCGTATTATTGCCACCAACCG  R: CAGCAACAGTCACAACAAACGC | 4 | 0.314 |
| B96 | Estoup et al, 1995 | F: GGGAGAGAAAGACCAAG  R: GATCGTAATGACTCGATATG | 12 | 0.784 |
| BTMS0081 | Stolle et al, 2009 | F: ACGCGCGCCTTCTACTATC  R: AGGGACACGCGAACAGAC | 2 | 0.599 |

1. **References**

Estoup, A., Solignac, M., Cornuet, J., Goudet, J., & Scholl, A. (1996). Genetic differentiation of continental and island populations of Bombus terrestris (Hymenoptera: Apidae) in Europe. Molecular Ecology, 5(1), 19-31. http://dx.doi.org/10.1111/j.1365-294x.1996.tb00288.x

Funk, C.R., Schmid-Hempel, R., & Schmid-Hempel, P. (2006). Microsatellite loci for Bombus spp. Molecular Ecology Notes, 6(1), 83-86. http://dx.doi.org/10.1111/j.1471-8286.2005.01147.x

Stolle, E., Rohde, M., Vautrin, D., Solignac, M., Schmid-Hempel, P., Schmid-Hempel, R., & Moritz, R. (2009). Novel microsatellite DNA loci for Bombus terrestris(Linnaeus, 1758). Molecular Ecology Resources, 9(5), 1345-1352. http://dx.doi.org/10.1111/j.1755-0998.2009.02610.x
