# Supplementary material for: Mating frequency estimation and its importance for colony abundance analyses in eusocial pollinators: a case study of Bombus impatiens (Hymenoptera: Apidae)
Source: J Econ Entomol. 2024 Aug 13;117(5):1712–22. doi: 10.1093/jee/toae178 (PMC11646103; doi:10.1093/jee/toae178)
Supplement: toae178_suppl_Supplementary_Materials [file toae178_suppl_supplementary_materials.zip › Supp.Material3_Birdetal.pdf]

Supplementary Methods

For "Mating frequency estimation and its importance ..." by Bird *et al.*

1 Haplodiploid multi-paternity model

For a microsatellite locus with k alleles in a given diploid individual, let G be the genotype and O be the phenotype (observed genotype). The genotyping error model of [Wang, 2004] – included for completeness – is,

$$\Pr(O = \{o_1, o_2\} \mid G = \{g_1, g_2\}, e) = \begin{cases} (1-e_2)^2 & \text{if } o_1 \in G \wedge o_2 = o_1 \\ 2\frac{e_2}{k-1}(1-e_2) & \text{if } o_1 \in G \wedge o_2 \notin G \text{ or } o_1 \notin G \wedge o_2 \in G \\ (2-\mathbb{I}[o_1 = o_2])(\frac{e_2}{k-1})^2 & \text{if } o_1 \notin G \wedge o_2 \notin G \end{cases} \quad (1)$$

if the genotype is homozygous ($g_1 = g_2$) and

$$\Pr(O = \{o_1, o_2\} \mid G = \{g_1, g_2\}, e) = \begin{cases} (1-e_2)^2 + (\frac{e_2}{k-1})^2 - 2e_1(1-e_2-\frac{e_2}{k-1})^2 & \text{if } o_1 \in G \wedge o_2 \in G \\ \frac{e_2}{k-1}(1-e_2) + e_1(1-e_2-\frac{e_2}{k-1})^2 & \text{if } o_1 \in G \wedge o_2 = o_1 \\ (2-\mathbb{I}[o_1 = o_2])(\frac{e_2}{k-1})^2 & \text{if } o_1 \notin G \wedge o_2 \notin G \\ \frac{e_2}{k-1}(1-e_2+\frac{e_2}{k-1}) & \text{otherwise} \end{cases} \quad (2)$$

if the genotype is heterozygous ($g_1 \neq g_2$). In equations 1 and 2, $e_1 \in (0, \frac{1}{2})$ is the probability that one allele in a heterozygous genotype is not observed in the phenotype (dropout error); and $e_2 \in (0, 1)$ is the probability that an allele is incorrectly observed in the phenotype as one of the other $k-1$ alleles (mistyping error). The indicator function $\mathbb{I}[\cdot]$ equals one if its argument is true and zero otherwise.

Let $O_0^{(\ell)}$ be the phenotype of the (diploid) mother, and let $O_i^{(\ell)}, i \in \{1 \ldots n\}$ be the phenotypes of the (diploid) offspring at a locus ℓ with k_ℓ alleles. If there are F haploid fathers and $p_i \in \{1 \ldots F\}$ indicates the father of the ith offspring, then the joint probability

of the observed phenotypes is

$$\begin{aligned}\Pr(O^{(\ell)} \mid p, e^{(\ell)}) = \\ \sum_{a=1}^{k_\ell}\sum_{b=1}^{k_\ell} \frac{1}{k_\ell^2}\Pr(O_0^{(\ell)} \mid G=\{a,b\}, e^{(\ell)}) \prod_{j=1}^{F}\sum_{c=1}^{k_\ell}\frac{1}{k_\ell}\times \\ \prod_{i=1}^{n} \mathbb{I}[p_i=j]\left(\frac{1}{2}\Pr(O_i^{(\ell)} \mid G=\{a,c\}, e^{(\ell)}) + \frac{1}{2}\Pr(O_i^{(\ell)} \mid G=\{b,c\}, e^{(\ell)})\right)\end{aligned} \quad (3)$$

which sums (integrates) over possible genotypes, assuming that these are equally probable *a priori* and obey Mendelian inheritance. If any individuals were not successfully phenotyped at a particular locus, then equation 3 is modified to sum over all possible phenotypes for that particular locus/individual.

We use a Bayesian approach for inferring the (unknown) paternity of offspring, and therefore need to place prior probabilities on possible assignments of offspring to fathers. We use a non-parametric prior, the so-called Chinese restaurant process [Pitman, 1995],

$$\Pr(p) = \frac{1}{\Gamma(n+1)}\prod_{j=1}^{F}\Gamma\left(\sum_{i=1}^{n}\mathbb{I}[p_i=j]\right).$$

This choice of prior encourages parsimonious paternity assignments (e.g. it favors assignments with fewer fathers and higher paternity skew), but allows for an arbitrary number of full-sibling groups as needed to explain the observed phenotypes. We place uniform priors on the genotyping error rates, $\Pr(e^{(\ell)}) \propto \mathbb{I}[0 < 2e_1^{(\ell)} < 1] \times \mathbb{I}[0 < e_2^{(\ell)} < 1]$. The unnormalized posterior distribution is thus,

$$\Pr(p, e \mid O) \propto \Pr(p)\prod_{\ell}\Pr(e^{(\ell)})\Pr(O^{(\ell)} \mid p, e^{(\ell)}). \quad (4)$$

2 Gibbs sampler

Here we describe a Gibbs sampler that targets the joint posterior in equation 4. We make use of data augmentation [Van Dyk and Meng, 2001] (e.g. introducing latent variables into the joint posterior) to derive the necessary conditional distributions.

2.1 Paternity assignments p and number of fathers F

Let $p^{(i\leftarrow j)}$ be a single-element modification of paternity assignments p so that

$$p_k^{(i\leftarrow j)} = \begin{cases} j & \text{if } k = i \\ p_k & \text{otherwise.}\end{cases}$$

Let $c_j = \sum_{k \neq i} \mathbb{I}[p_k^{(i \leftarrow j)} = j]$ be counts of offspring with paternity assignment j, excluding the ith offspring. Following algorithm 4 in [Neal, 2000], the paternity for offspring i is sampled according to

$$\Pr(p_i = j \mid p_{-i}, e, O) \propto \begin{cases} c_j n^{-1} \prod_\ell \Pr(O^{(\ell)} \mid p^{(i \leftarrow j)}, e^{(\ell)}) & \text{if } c_j > 0 \\ n^{-1} \prod_\ell \Pr(O^{(\ell)} \mid p^{(i \leftarrow j)}, e^{(\ell)}) & \text{if } c_j = 0 \wedge j = j^* \\ 0 & \text{otherwise,} \end{cases}$$

where j^* is the lowest positive integer j such that $c_j = 0$. Thus, the values of p always span contiguous integers starting from 1, and the number of fathers is always $F = \max p$.

2.2 Genotyping error rates

To sample the dropout and mistyping error rates $e_1^{(\ell)}$ and $e_2^{(\ell)}$, we augment the model with latent variables that are the true genotypes and counts of error events. Let $G_0^{(\ell)}$ and $G_i^{(\ell)}, i \in \{1 \dots n\}$ be the diploid maternal and offspring genotypes, and let $g_j^{(\ell)}, j \in \{1 \dots F\}$ be the haploid paternal genotypes. Let $\epsilon_{i,1}^{(\ell)}$ and $\epsilon_{i,2}^{(\ell)}$, $i \in \{0 \dots n\}$, be counts of dropout and mistyping errors that result in a genotype $G_i^{(\ell)}$ being observed as a phenotype $O_i^{(\ell)}$. Error events do not exist for haploid fathers, which have no phenotypes in our model – but if fathers or haploid offspring were phenotyped, only mistyping errors would be possible.

The maternal genotype is sampled according to probabilities,

$$\Pr(G_0^{(\ell)} = \{a, b\} \mid p, e^{(\ell)}, O^{(\ell)}) \propto \Pr(O_0^{(\ell)} \mid G = \{a, b\}, e^{(\ell)}) \prod_{j=1}^{F} \sum_{c=1}^{k_\ell} \frac{1}{k_\ell} \times$$
$$\prod_{i=1}^{n} \mathbb{I}[p_i = j] \left(\frac{1}{2} \Pr(O_i^{(\ell)} \mid G = \{a, c\}, e^{(\ell)}) + \frac{1}{2} \Pr(O_i^{(\ell)} \mid G = \{b, c\}, e^{(\ell)}) \right)$$

for $1 \leq a, b \leq k_\ell$. Conditional on the maternal genotype, the paternal genotypes are sampled according to probabilities,

$$\Pr(g_j^{(\ell)} = c \mid p, e^{(\ell)}, O^{(\ell)}, G_0^{(\ell)} = \{a, b\}) \propto$$
$$\prod_{i=1}^{n} \mathbb{I}[p_i = j] \left(\frac{1}{2} \Pr(O_i^{(\ell)} \mid G = \{a, c\}, e^{(\ell)}) + \frac{1}{2} \Pr(O_i^{(\ell)} \mid G = \{b, c\}, e^{(\ell)}) \right)$$

for $1 \leq c \leq k_\ell$. Conditional on parental genotypes, the offspring genotypes are sampled according to probabilities,

$$\Pr(G_i^{(\ell)} = x \mid e^{(\ell)}, O^{(\ell)}, G_0^{(\ell)} = \{a, b\}, g_{p_i}^{(\ell)} = c) \propto$$
$$\begin{cases} \Pr(O_i^{(\ell)} \mid G = x, e^{(\ell)}) & \text{if } x = \{a, c\} \text{ or } x = \{b, c\} \\ 0 & \text{otherwise.} \end{cases}$$

As in equation 3, missing phenotype data is accomodated by summing conditional genotype probabilities over possible phenotypes. Counts of error events are sampled conditional on genotypes and phenotypes. If genotype $G_i^{(\ell)} = \{g_1, g_2\}$ is homozygous then discrepancies with phenotype $O_i^{(\ell)} = \{o_1, o_2\}$ can only be explained by a single sequence of mistyping errors, and thus

$$\epsilon_i^{(\ell)} = \begin{cases} \{0,0\} & \text{if } o_1 \in G \wedge o_2 \in G \\ \{0,1\} & \text{if } o_1 \in G \wedge o_2 \notin G \text{ or } o_1 \notin G \wedge o_2 \in G \\ \{0,2\} & \text{if } o_1 \notin G \wedge o_2 \notin G. \end{cases}$$

However, if the genotype is heterozygous then multiple combinations of events could result in the same phenotype. The probabilities depend upon the type of mismatch:

$$\Pr(\epsilon_i^{(\ell)} = x \mid e^{(\ell)}, O_i^{(\ell)}, G_i^{(\ell)}) =$$

$$\begin{cases} (1-2e_1)(1-e_2)^2 & \text{if } x = \{0,0\} \\ (1-2e_1)(\frac{e_2}{k_\ell - 1})^2 & \text{if } x = \{0,2\} \\ 4e_1 \frac{e_2}{k_\ell - 1}(1-e_2) & \text{if } x = \{1,1\} \\ 0 & \text{otherwise} \end{cases} \quad (\text{when } o_1 \in G \wedge o_2 \in G)$$

$$\begin{cases} e_1(1-e_2)^2 & \text{if } x = \{1,0\} \\ (1-2e_1)(1-e_2)\frac{e_2}{k_\ell - 1} & \text{if } x = \{0,1\} \\ e_1(\frac{e_2}{k_\ell - 1})^2 & \text{if } x = \{1,2\} \\ 0 & \text{otherwise} \end{cases} \quad (\text{when } o_1 \in G \wedge o_2 = o_1)$$

$$\begin{cases} (1-2e_1)(\frac{e_2}{k_\ell - 1})^2 & \text{if } x = \{0,2\} \\ 2e_1(\frac{e_2}{k_\ell - 1})^2 & \text{if } x = \{1,2\} \\ 0 & \text{otherwise} \end{cases} \quad (\text{when } o_1 \notin G \wedge o_2 \notin G)$$

$$\begin{cases} (1-2e_1)(1-e_2)\frac{e_2}{k_\ell - 1} & \text{if } x = \{0,1\} \\ (1-2e_1)(\frac{e_2}{k_\ell - 1})^2 & \text{if } x = \{0,2\} \\ 2e_1(1-e_2)\frac{e_2}{k_\ell - 1} & \text{if } x = \{1,1\} \\ 2e_1(\frac{e_2}{k_\ell - 1})^2 & \text{if } x = \{1,2\} \\ 0 & \text{otherwise} \end{cases} \quad (\text{for all other cases})$$

Finally, conditional on counts of genotype errors, the genotyping error rates follow beta distributions: these have shape parameters $\alpha_1 = 1 + \sum_{i=0}^{n} \epsilon_{i,1}^{(\ell)}$, $\beta_2 = 1 + h_\ell - \sum_{i=0}^{n} \epsilon_{i,1}^{(\ell)}$ for $2e_1^{(\ell)}$; and $\alpha_2 = 1 + \sum_{i=0}^{n} \epsilon_{i,2}^{(\ell)}$, $\beta_2 = 1 + 2m_\ell - \sum_{i=0}^{n} \epsilon_{i,2}^{(\ell)}$ for $e_2^{(\ell)}$. The quantities m_ℓ and h_ℓ are the total numbers of nonmissing phenotypes and heterozygous genotypes (with nonmissing phenotype) for locus ℓ.

3 Implementation

The Gibbs sampler (and some helper functions) are implemented in an R package at `https://github.com/nspope/paternityDP`. The code in Figure 1 illustrates the generation of Markov chain Monte Carlo samples for one of the colonies analyzed in the main text.

References

[Neal, 2000] Neal, R. M. (2000). Markov chain sampling methods for Dirichlet process mixture models. *Journal of computational and graphical statistics*, 9(2):249–265.

[Pitman, 1995] Pitman, J. (1995). Exchangeable and partially exchangeable random partitions. *Probability theory and related fields*, 102(2):145–158.

[Van Dyk and Meng, 2001] Van Dyk, D. A. and Meng, X.-L. (2001). The art of data augmentation. *Journal of Computational and Graphical Statistics*, 10(1):1–50.

[Wang, 2004] Wang, J. (2004). Sibship reconstruction from genetic data with typing errors. *Genetics*, 166(4):1963–1979.

```
remotes::install_github("nspope/paternityDP")
library(paternityDP)

genotype_data <- genotype_array_from_txt(
  system.file("example/colony2_genotypes.txt", package="paternityDP")
)

#drop loci w/ >50% missing loci
genotype_data <- remove_loci_with_excessive_missingness(
  genotype_data, 0.5
)

#drop samples w/ >50% missing, retain samples with "Qu" in name
genotype_data <- remove_samples_with_excessive_missingness(
  genotype_data, 0.5, always_keep = "Qu"
)

#drop loci w/ 1 allele
genotype_data <- remove_monomorphic_loci(genotype_data)

#find queen index (sample name with "Qu" in it)
the_queen <- grep("Qu", colnames(genotype_data))

#fit model
fit <- sample_paternity_and_error_rates_from_joint_posterior(
  genotype_data,
  mother=the_queen,
  number_of_mcmc_samples=1000,
  global_genotyping_error_rates=FALSE, #use same rate across loci?
  add_unsampled_allele=FALSE #include an unobserved allele per locus?
)

#outputs
fit$paternity #columns are MCMC samples, rows are individuals
fit$dropout_error #columns are MCMC samples, rows are loci
fit$mistyping_error #columns are MCMC samples, rows are loci

#posterior probabilities for number of paternities
table(fit$number_of_fathers) / length(fit$number_of_fathers)
```

Figure 1: R code illustrating application of the described Gibbs sampler on a single colony.
