# Supplementary material for: Mating frequency estimation and its importance for colony abundance analyses in eusocial pollinators: a case study of Bombus impatiens (Hymenoptera: Apidae)
Source: J Econ Entomol. 2024 Aug 13;117(5):1712–22. doi: 10.1093/jee/toae178 (PMC11646103; doi:10.1093/jee/toae178)
Supplement: toae178_suppl_Supplementary_Materials [file toae178_suppl_supplementary_materials.zip › Supp4_Birdetal.pdf]

Supplementary Materials 4

For "Mating frequency estimation and its importance ..." by Bird et al.

1. Error Estimation Code, used to estimate dropout and mistyping error for each colony:

```
install.packages("ggplot2")

install.packages("devtools")

library(ggplot2)

library(sydneyPaternity)

library(devtools)

setwd("~/Desktop/bombus_data")

devtools::install_github("nspope/sydneyPaternity")


# 1. Load the genotype data we just saved into an array

all_genotype_data <- genotype_array_from_txt("prelim_genotype_data_forR.txt")

all_genotype_data #look and make sure names are OK



sum(all_genotype_data == 0 | is.na(all_genotype_data)) / prod(dim(all_genotype_data))


##############Colony ########################

genotype_data <- genotype_array_from_txt("colony.txt")

genotype_data


# 2. Jointly estimate paternity and error rates
```

```
set.seed(1)

mcmc_paternity_f <-

  sample_paternity_and_error_rates_from_joint_posterior(genotype_data,

                                                        mother = 1,

                                                        number_of_mcmc_samples=1000)


# 3. Visualize joint posterior distribution of error rates/paternity

plot_posterior(mcmc_paternity_f)


# 4. Visualize dropout and mistyping errors

plot_genotyping_errors(mcmc_paternity_f, genotype_data)


#5. pull out error rates

mean(mcmc_paternity_f$dropout_rate)

mean(mcmc_paternity_f$mistyping_rate)
```

2. Table of Error Results Per Colony:

Colony	Dropout	Mistyping
1	0.0041	0.0038
2	0.0036	0.0027
3	0.0033	0.0033
4	0.0070	0.0070
5	0.0059	0.0175
6	0.0094	0.0115
7	0.0025	0.0075
8	0.0091	0.0044
9	0.0085	0.0193
10	0.0243	0.0151
11	0.0189	0.0060

12	0.0167	0.0063
13	0.0282	0.0185
14	0.0173	0.0172
15	0.0141	0.0166
16	0.0127	0.0092
17	0.0070	0.0058
18	0.0155	0.0049
19	0.0044	0.0116
20	0.0114	0.0129
21	0.0094	0.0104
22	0.0161	0.0167
23	0.0093	0.0095
24	0.0097	0.0142
25	0.0206	0.0068
26	0.0066	0.0040
27	0.0050	0.0040
28	0.0160	0.0093
29	0.0100	0.0476
30	0.0044	0.0042
Range	0.002548654	0.002733194
	0.02818944	0.04760292
Median	0.0094	0.0092
