# Supplementary material for: Mating frequency estimation and its importance for colony abundance analyses in eusocial pollinators: a case study of Bombus impatiens (Hymenoptera: Apidae)
Source: J Econ Entomol. 2024 Aug 13;117(5):1712–22. doi: 10.1093/jee/toae178 (PMC11646103; doi:10.1093/jee/toae178)
Supplement: toae178_suppl_Supplementary_Materials [file toae178_suppl_supplementary_materials.zip › Supp5_Birdetal .pdf]

Supplementary Materials 5

For “Mating frequency estimation and its importance ...” by Bird et al.

Summary table of the number of offspring included in microsatellite analysis per colony, the PCR plate IDs of all members of each colony, the number of Loci removed for each colony and the number of individual samples removed. Number of individuals removed includes floaters removed and those removed due to insufficient data.

Colony	Number of Offspring	PCR Plate #	Number of Loci Removed	Number of Samples Removed
1	27	2,1	4	1
2	32	2,1	4	0
3	25	2,1	4	1
4	25	2,1	4	0
5	22	2,1,3	3	2
6	26	2,3	3	2
7	32	2,3	3	0
8	31	3	4	2
9	30	2,3,4	4	5
10	31	2,4	3	0
11	29	2,4	3	5
12	28	2,4	3	6
13	19	4,9	8	3
14	13	4,9	3	2
15	20	4,5	4	8
16	19	5	2	2
17	20	5	4	1
18	20	5	1	2
19	20	5	3	2
20	17	5,6	3	1
21	20	6	2	3
22	16	6	3	3
23	20	6	3	1
24	21	6,9	5	4
25	14	6,7	2	2
26	20	7	2	1

27	21	7	3	2
28	20	7	1	5
29	20	7	1	7
30	21	8	3	1
